# Processing-Dependent Releasing of Iron from Plant Ferritin in Cereal-Based Foods Designed for Iron Delivery in Inflammatory Bowel Disease

**DOI:** 10.3390/molecules31030510

**Published:** 2026-02-02

**Authors:** Magdalena Zielińska-Dawidziak, Agnieszka Makowska, Magdalena Czlapka-Matyasik, Aleksandra Proch, Przemysław Niedzielski

**Affiliations:** 1 Department of Food Biochemistry and Analysis, Poznań University of Life Sciences, Mazowiecka 48, 60-624 Poznan, Poland; 2 Department of Food Technology of Plant Origin, Poznań University of Life Sciences, Wojska Polskiego 31, 60-624 Poznan, Poland; agnieszka.makowska@up.poznan.pl; 3 Department of Human Nutrition and Dietetics, Poznań University of Life Sciences, Wojska Polskiego 31, 60-624 Poznan, Poland; magdalena.matyasik@up.poznan.pl; 4 Department of Analytical Chemistry, Faculty of Chemistry, Adam Mickiewicz University, 89b Umultowska Street, 61-614 Poznan, Poland; aleksandra.proch@amu.edu.pl (A.P.);

**Keywords:** ferritin iron, ferrous iron, ferric iron, inflammatory bowel diseases, food designing

## Abstract

Fortified soybean sprouts have been proposed as a source of ferritin-iron in food for the treatment of anemia in inflammatory bowel disease. Eight products with the addition of the sprouts have been designed, and iron speciation was studied in them by flame atomic absorption spectrometry (total iron content) and spectrophotometry (ionic forms). Non-ionic iron content, considered ferritin-iron content, was calculated as the difference between total and inorganic iron content. The production of crispbread disrupted ferritin and caused the release of ferritin-iron. A loss of ~3% of ferritin-iron was noted in rice wafers containing a coarse fraction of sprouts, and 0–10% in instant products (‘kisiel’, ‘budyn’, and groats). Lost ferritin-iron was converted mostly into ferrous iron, except for crispbread, in which Fe(III) constituted ~30%. The designed products are valuable sources of iron, with a high content of plant ferritin.

## 1. Introduction

Iron deficiency (ID) is the most common form of malnutrition globally; its effects are critical for human performance and development. ID is responsible for most anemia cases, but also for low immunity and abnormal mental development [1,2,3]. The problem may be reduced by food enrichment or fortification, and this strategy is recommended by the governments of many countries and international organizations. However, iron introduction into food may have undesirable effects on foodstuffs’ quality, changing their physicochemical and sensorial properties, while food processing may strongly decrease iron bioavailability [4,5]. For this reason, iron is often encapsulated to limit the iron ions’ contact with food ingredients, to stop oxidation and discoloration, and to increase iron stability [3,4].

The promise is the application of biofortified plants, e.g., ferritin-containing preparations. Ferritin is a natural, spherical protein complex that stores thousands of iron ions in its core. It is considered a highly bioavailable form of iron [3,6,7]. The sprouts of *leguminaceae* growing in abiotic stress conditions induced by high concentrations of Fe(II) ions accumulate iron in this protein. Nevertheless, it is necessary to maintain the stability of ferritin during food processing, because the protein nano-cage is sensitive to both temperature and pH modification. The isolated protein tolerates temperatures up to 80 °C and pH in the range of 3–8, but as a food ingredient, when the food matrix protects its molecule, it may survive food processing [7,8]. It is important to keep the iron in that form during food processing because of a different way of its absorption. It is proven that ferritin iron is slowly taken up independent of heme iron and the ferrous form [9]. Therefore, it is proposed for individuals who do not tolerate traditional iron supplements or are suffering from inflammatory bowel diseases in remission. The presented study aimed to design different types of food fortified with ferritin iron from fortified soybean sprouts and compare the iron forms in the prepared foodstuffs. Thus, the research included a speciation analysis of iron, which allowed the assessment of ferritin iron content in the designed products. To our knowledge, this is the first study that attempts to design functional foods for the very demanding group of patients suffering from iron deficiency anemia.

## 2. Results and Discussion

The study’s first aim was to design various cereal products fortified with iron for patients with inflammatory bowel diseases (IBD, encompassing Crohn’s disease and ulcerative colitis). Cereal products constitute an important part of the human diet. Their diversity allows the design of several foodstuffs (lunch and breakfast products, snacks, and desserts) that can serve as a source of additional iron for IBD patients. Due to the slow release of iron from ferritin and a different mechanism of ferritin-iron absorption, it has been suggested that ferritin sources may be a promising alternative to traditional iron supplements. However, IBD patients require special attention in providing well-absorbed iron since the disease is associated with a significant loss of iron and problems in its assimilation [10,11]. The diet of these patients requires many other restrictions related to, among others, dairy products, including lactose, the supply of soluble fiber, and anti-inflammatory components. The patients’ complaints, in addition to anemia, are associated with dysbiosis, diarrhea, gluten intolerance, and other nutritional deficiencies [12,13].

As mentioned above, sources rich in ferritin, such as fortified soy sprouts (FSS), may be beneficial for patients who do not tolerate traditional iron supplements [9,14,15]. FSS in the experiment contained 560.6 Fe mg/100 g of dry matter (515.8 mg in the fresh material). The FSS addition was adjusted so that the consumed portion of the product provided an additional 5 mg of iron compared with the non-fortified product. According to the applicable levels in Poland [16], the obtained fortified products meet the requirements for the supply of iron for fortified food. The iron content complied with EU regulations [17].

To diversify the diet of patients, foodstuffs that can be an element of different meals were designed: breakfast products (bread and crispbread), lunch products (pasta and groats), instant desserts (‘kisiel’ and ‘budyn’), and snacks (rice wafers and corn snacks). Consumers can include them in their diet at different times of the day, with the assumed consumption of four different products per day. The products can provide an additional 20 mg of iron each day. The products’ convenience, easy digestion, and diversity are important to encourage patients to consume the same product in several doses per day for a long time. Efforts were made to limit the portion size and simultaneously to maximize the addition of FSS. Technological limitations and those resulting from consumer acceptance have been considered when producing the proposed food.

All products were designed according to recommendations for integration into a sustainable diet for subjects with Inflammatory Bowel Disease (IBD). Products were adjusted to meet nutritional needs, expected health status (remission), iron reserves, low-FODMAP, lactose-free, and energy requirements, according to recommendations for IBD [18]. Kisiel and budyn (with lactose-free milk) are easily digestible desserts. Recommended for individuals with increased energy needs, reduced appetite, or gastrointestinal sensitivity. Groats were recommended as staple carbohydrate sources in balanced meals, providing complex carbohydrates and dietary fiber, suitable for regular consumption [19,20]. Pasta was recommended as a base for main meals when combined with vegetables and protein sources. Portion control was advised to maintain appropriate energy intake. Gluten-free bread was advised for consumption by subjects with gluten intolerance or celiac disease (often recommended for IBD subjects) and was not recommended as a replacement for main meals [21]. We designed also rice wafers, which were labelled as snacks or bread substitutes. They were labelled as best paired with protein or fat sources to improve satiety and glycemic response [22]. The corn snacks have been allocated for occasional consumption as snacks, with the remark that they should not replace core meals.

Ultimately, it was possible to produce a range of products following the recipe composition and technologies presented above. The analyzed composition of the products and their energy value are presented in Table 1.

The stability of isolated ferritin and that which is present in the food matrix differs [7,23,24]. So, apart from designing foodstuffs, the experiment aimed to evaluate the speciation of iron in the obtained products, and this issue is the focus of the discussion.

The ingredients of the designed products provided in the recipe serve a technological function, influencing the nutritional value of the foodstuffs. They are sources of important ingredients in the diet of patients suffering from IBD. The soluble fiber content in the designed products increased by adding technological components, i.e., inulin, pectin, or guar gum. To increase the antioxidant activity (AA) of the foodstuffs, dried vegetables (in groats), buckwheat flour (bread), or ascorbic acid (‘kisiel’) were used. AA also results from the addition of FSS. Among all designed products, the groats AA is the highest, compared with other products, due to the addition of dried vegetables.

Due to the variable bioavailability of iron from food, depending on the iron form, the occurrence of iron forms in the designed product is important.

Anticipating this, we emphasize that in FSS iron can be found mainly as ferritin-iron and in ionic form as Fe(II). The calculated ferritin-iron content in FSS was ~420.5 mg/100 g d.m., which represents ~75% of the total iron content in the prepared sprouts (Table 2). During the production of the designed food, ferritin-iron may be released from the protein and present in the final product as an ionic form (Fe(II) or Fe(III)) due to ferritin denaturation. It can affect its absorption in the intestine.

DMT1 (divalent metal ion transporter 1) transports Fe(II) together with a proton across the brush border membrane of the proximal small intestine. It is speculated that this form is taken up in 5–15% [25]. Currently, it is known that Fe(III) can also be absorbed directly from the intestinal lumen. This system is dependent on beta 3-integrin. The limitation of this route efficiency results from Fe(III)’s restricted solubility in neutral and alkaline pH. Thus, citrate and organic acids may increase its solubility. Whereas cytochrome b from the brush border of enterocytes helps Fe(III) reduction and consequently its absorption by DMT1 [26]. Ferric iron is absorbed in 2–10%. In the case of ferritin, a distinct route of iron absorption exists. It is receptor-mediated endocytosis. Due to these different mechanisms, ingested ionic or heme iron does not slow ferritin iron absorption, which is at a level higher than 15% from food [9].

Thus, it was important to determine iron speciation after designing the products.

In the case of desserts, all determined forms of iron (Fe(II), Fe(III), and ferritin-iron) were detected. Not-fortified desserts (NF) differed in the content of non-ionic iron. ‘Budyn’, which contains 20% powdered milk, had a higher content of non-ionic iron form. Thus, not-fortified ‘budyn’ contained 3.4–4.0 mg of total iron/100 g (sum of Fe(II), Fe(III), and non-ionic Fe), while ‘kisiel’ contained 0.5–1.6 mg of total iron/100 g. As a result of the dessert treatment with boiling water, the loss of non-ionic iron of about 10% was observed in the ‘budyn’, although it was not recorded in ‘kisiel’ (Table 2). If the loss of non-ionic iron was observed, then Fe(II) content increased. However, in the case of ‘kisiel’, the formation of an Fe(III) chelate might have occurred with added ascorbic acid, which may increase iron absorption.

In the group of lunch products, instant groats with vegetables and pasta were prepared. During the groats preparation for consumption (mixing with hot water), an average decline of 7% of non-ionic iron and its conversion to Fe(II) was observed, and the content of Fe(III) slightly increased (Table 2).

Pasta production causes minor ferritin-iron losses, which were not large (~3%). However, pasta cooking significantly decreased non-ionic iron, reducing it by 32–39% depending on cooking time. An important fact is that the iron did not leak from the product. There was no iron found in the water in which the pasta was cooked. It was released from ferritin and was still present in the product, but in the ionic form of Fe(II) (Table 2).

In one of the breakfast products, crispbread, there was a complete breakdown of ferritin, and non-ionic iron was converted into the least absorbable form, Fe(III) (Table 2). Moreover, an exceptionally strong, undesirable grassy taste appeared (data not published). Thus, the crispbread was excluded from the compositional studies. The observed changes must have resulted from the applied high pressure and temperature during extrusion [8,27].

In gluten-free bread, the ferritin-iron loss was 25–30%, and the released iron was present in the product as Fe(II). It was also found that ferritin losses were lower when the bread was prepared with sourdough than with the addition of yeast. However, the differences are not statistically significant.

The decrease in complexed iron may result from the presence of bacterial and yeast proteolytic enzymes [28], but also temperature action. Although in vitro studies indicate that the release of iron from ferritin can begin at around pH 5 [29] due to the structural destabilization during fermentation [30], we did not observe extensive iron release from ferritin during sourdough fermentation.

Surprisingly, this protein is considered quite stable during baking, because inside the bread the temperature does not exceed 100 °C [20,21], and the food matrix also influences iron bioaccessibility [7].

The last designed products were goods classified as snacks: corn snacks and rice wafers. They were produced with the application of coarse and fine fractions of FSS. In the case of corn snacks, the loss of ferritin was high, reaching 27–35%. Iron released from ferritin was present as the ferrous form. Lower losses were recorded when FSS with larger rather than finer granulation was added. In this larger fraction, where ferritin was present in not-destroyed plant cells, it was less susceptible to temperature-dependent denaturation.

In rice wafers, the losses of ferritin were lower, 3–4%. We noted that in the case of a fine fraction of FSS addition, some ferritin-iron was released and oxidized, and an increase in ferric form was noted in the final product.

The presented study should be perceived as a preliminary study. It needs to be continued and followed by the study on in vitro and in vivo bioavailability, clinical studies, research on long-term stability, surveys on patient acceptance, cost-effectiveness analyses of these products, etc.

## 3. Materials and Methods

### 3.1. FSS Preparation

Soybean seeds (*Glycine max*, Aldana var.) were obtained from the Department of Genetics and Plant Breeding, Poznan University of Life Sciences. The sprouts were cultured in a climate chamber (ADAPTIS, Conviron, Winnipeg, MB, Canada) for 7 days at room temperature, with controlled humidity and illumination. They were watered every day with fresh 20 mM FeSO_4_ (Chempur, Piekary Śląskie, Poland) solution. The obtained sprouts were dried to 8–10% moisture in the oven, then milled or cut, and stored in tightly closed containers at 4 °C.

### 3.2. Designed Products

The products were designed to deliver an additional 5 mg of iron to the diet with one portion.

#### 3.2.1. Instant Sweet Desserts

##### Kisiel

Polish desert, of the jelly type, consisted of pregelatinized potato starch (52.6%), inulin (26.8%), maltodextrin (14.6%), milled FSS (3.6%), ascorbic acid (1.0%), fruit flavor (apricot, orange, peach, or apple; 0.8%), food dye (0.3%), *β*-carotene (0.1%), and sucralose (0.1%). The dry kisiel concentrate with ferritin was packed 27 g each in heat-sealable bags made of a triplex PET/AL/PE laminate.

##### Budyn

Dessert close to pudding and cherry and vanilla flavored, consisted of pregelatinized potato starch (36.4%), inulin (22.7%), skim lactose-free milk powder (20.3%), whole lactose-free milk powder (16.0%), maltodextrin (1.5%), milled FSS (2.1%), cherry or vanilla flavor (0.6%), food dye (0.3%), *β*-carotene (0.1%), and sucralose (0.1%). Chocolate budyn consisted of pregelatinized potato starch (35.6%), inulin (22.1%), skim milk powder (19.8%), whole milk powder (15.6%), maltodextrin (1.4%), milled soy sprouts containing ferritin (2.1%), cocoa powder (3.0%), food dye (0.3%), *β*-carotene (0.1%), and sucralose (0.1%). The produced dry budyn concentrate with ferritin was packed 45 g each in heat-sealable bags made of a triplex PET/AL/PE laminate.

To prepare the desserts for consumption, the bag’s contents were poured into a cup, 175 mL of boiling water was added, and it was stirred for 1 min.

#### 3.2.2. Lunch Products

##### Groats

The dry instant groats consisted of instant wheat–corn groats (80.6%) and additives such as pregelatinized potato starch (3.1%), dried vegetables (mix of carrots, parsnips, celery, onion, leek, parsley, 8.8%), coffee whitener (4.0%), salt (1.0%), yeast extract (0.7%), and maltodextrin (0.2%). Unit portions of the dry food concentrate (62 g) were packed in bags. Milled FSS (1.6%) were packed separately. Bags were made of heat-sealable barrier laminate.

The instant groats were prepared for consumption by pouring the instant product with 150 mL of boiling water and mixing. After ~5 min, FSS was added to the ready-to-eat groats.

##### Pasta

Pasta enriched in ferritin was prepared from durum wheat semolina (92.6%), vital wheat gluten (2%), inulin (0.5%), fresh eggs (2% as a dry mass), milled FSS (1.9%), and water (dough moisture 32%). The dough was mixed for 20 min in a pasta extruder of Italpast type P-120 (Fidenza, Parma, Italy) and then extruded through Teflon-coated dies, giving it the shape of ribbons. The extruded pasta was placed on sieves and dried in a stationary dryer at a temperature of 40 °C for 9–10 h, that is, until the final moisture reached 12% ± 0.5%. After drying, the pasta was left for 4 h in the chamber at ambient temperature for stabilization and then packed in proprietary cellophane unit packages (100 g). Preparing the product for consumption consisted of cooking pasta in tap water for 5 or 7 min. Then, 200 mL of cold water was poured into the pot, and the pasta was drained in a colander. The pasta was ready to eat after 1 min.

#### 3.2.3. Breakfast Products

##### Gluten-Free Bread

Bread consisted of buckwheat flour (17.0%), rice flour (17.0%), corn flour (17.0%), potato flour (28.1%), potato grits (4.2%), ground milled FSS (3.2%), salt (0.8%), sugar (5.1%), guar gum (0.8%), pectin (2.1%), rapeseed oil (2.5%), and water (dough consistency 160). Two different technologies were used for bread preparation: the addition of yeast (4.2%) or sourdough culture starter LV1 (0.5%). Dry ingredients were thoroughly mixed in the bowl of the mixer (KitchenAid Artisan Heavy Duty, Benton Harbor, MI, USA). For sourdough preparation, a 40% dry ingredients mixture was blended with water and LV1 culture starter and placed in a fermentation chamber (temperature, 30 °C; 12 h). The sugar, salt, and yeast or sourdough were dissolved in water and added to the dry ingredients. The dough was mixed for 6 min, removed to the bread molds, and fermented in a fermentation chamber in the following conditions: RH 70%, temperature 30 °C for 40 min. Then, the molds were placed in the oven (MIWE condo, GETH, Kraków, Poland) and baked at 230 °C for 30 min. After this time, the molds were removed from the oven, the loaves were cooled down, cut into slices, packed (100 g, 4 pcs) in PP bags, and frozen.

##### Crispbread

Crispbread was produced by the extrusion-cooking method in a corotating twin-screw extruder (BC-45, Clextral, Firminy, France) from the triticale grits (88.5%, particle size 0.5–2.0 mm, moisture content 12%), corn grits (8.8%), salt (0.8%), and cut FSS (1.9%, particle size 350 µm). In the first step, the sprouts were mixed with part of the triticale grits in a ratio of 1:10. Next, the premixes were blended for 10 min with the remaining part of the triticale grits in a mixer and directly transported to an extruder. The temperature of the process was 130–180 °C [31]. After extrusion, the pieces of crispbread were allowed to cool and packed in PP unit packages (50 g, 5 pcs).

#### 3.2.4. Snacks

##### Rice Wafers

Wafers were prepared from husked brown rice grain (98.1%) and FSS (1.9%). The FSS were cut into particles with sizes of 3.0–1.5 mm (coarse fraction) and 1.5–0.5 mm (fine fraction). Rice grain was moistened to 16–17% water content and left for 24 h to equilibrate the moisture. Immediately before production, the rice and FSS (coarse or fine fraction) were thoroughly blended. The portion of the mixture was automatically weighed and placed in the forming element of the rice wafer machine and trapped in it for 4–8 s at temperatures of 190–250 °C. Then, the wafers were allowed to cool and packed in PP unit packages (100 g, 10 pcs).

##### Corn Snacks

These snacks were prepared as was described previously [8,27]. The corn snacks were prepared from the corn grits (96.1%) and mixed with the cut FSS (coarse or fine fraction). The addition of FSS was 3.9%. All ingredients were blended thoroughly for 20 min, and then the blend was put in a plastic container and closed. The extrusion was conducted at single-screw extruder TS-45 (Metalchem, Gliwice, Poland) (L:D 12, compression ratio of 3:1, nozzle diameter 3 mm) at a temperature of 140 °C. The programmed temperature was the same in all three zones of the barrel. After extrusion, the snacks were allowed to cool and packed in PP bags (25 g).

### 3.3. The Energy Value and Nutrient Profile of Designed Products

The energy value of the products and total carbohydrate content were determined according to Regulation 1169/2011 [17]. Protein content with the Kjeldahl method [32], fat content with Soxhlet method [33], and fiber content [34] were also analyzed. Antioxidant activity was determined with the ORAC method [35].

### 3.4. Iron Content Determination

FSS were mineralized and subjected to the total iron content (determined by atomic absorption spectroscopy (λ = 248.3, gap width of 0,15 nm) and ionic iron content (spectrophotometrically, by thiocyanate (λ = 470 nm) and 2 2′ bipyridyl (λ = 520 nm) reactions) in the environment of HCl (pH < 2). Complexed iron content, considered ferritin iron content, was calculated as the difference between total and inorganic iron content [36].

The iron content in the products was determined after their preparation for consumption, which was followed by freeze-drying. In the case of pasta, iron content in the dry product, cooked product, and water used for pasta cooking was analyzed.

### 3.5. Statistical Analysis

All analyses were performed in seven replicates, and the results were expressed as the average value ± standard deviation. If it were possible, significant statistical differences in iron stability were studied using the Student’s *t*-test with Statistica software (v.13.3., Tibco Software Inc., Palo Alto, CA, USA).

## 4. Conclusions

A range of products enriched with sprouts containing non-ionic iron in the form of ferritin was developed, dedicated to people suffering from iron deficiency, including patients with inflammatory bowel diseases. The products were designed to be easily digested and convenient for consumption: instant or ready-to-eat goods. The stability of ferritin in the tested products varied depending on the production process used and the preparation of the product for consumption. The greatest losses of ferritin were noted in crispbread (100%), and a slight loss was noted in the case of rice wafers produced using the coarse FSS fraction (3%), and instant products (‘kisiel’, ‘budyn’, and groats, in the range of 0–10%). All the designed products were good sources of iron, and despite these losses of ferritin, they were still good sources. Beyond the crispbread, ferritin iron released during the process goes in quite well in the absorbable form Fe(II). Our research suggests that the simultaneous exposure to high temperature and pressure, even for a short period of time, leads not only to ferritin degradation but also to the oxidation of iron to Fe(III), as observed in crispbread and rice cakes. This study demonstrates that with appropriate control of processing conditions and matrix composition, it is possible to maintain significant iron content in potentially beneficial forms and limit ferritin degradation in cereal products.

## 5. Patents

Zielińska-Dawidziak M., Twardowski T. Method for manufacturing a composition with increased content of plant ferritin and other forms of iron, the composition, and its use in manufacturing a preparation for supplementing the human diet. Pat. PL218747

Przygodzki R., Zielińska-Dawidziak M., Remiszewski M., Langner R., Makowska A., Piasecka-Kwiatkowska D., Dry, dietary concentrates of instant meals with groats or pasta, especially supporting nutrition in anemia, and a method for their manufacture. Pat.PL227146

Przygodzki R., Zielińska-Dawidziak M., Remiszewski M., Kupka A., Makowska A., Piasecka-Kwiatkowska D., Dry, dietary instant dessert concentrates, particularly those supporting nutrition in anemia, and a method for their production. Pat PL.227148

Przygodzki R., Jeżewska M., Błasińska I., Zielińska-Dawidziak M., Piasecka-Kwiatkowska D., Makowska A. Dry, dietary instant soup concentrates, particularly those supporting nutrition in anemia, and a method for their production. Pat. PL227147

Przygodzki R., Kulczak M., Korbas E., Zielińska-Dawidziak M., Piasecka-Kwiatkowska D., Makowska A. Dry, dietary instant cereal breakfast concentrates, particularly those supporting nutrition in anemia, and a method for their production. Pat PL.227145

## Figures and Tables

**Table 1:** Compositional data of the designed products and their antioxidant activity.

Product	Energy (kJ/kcal)	Total Protein [g/100 g]	Total Fat [g/100 g]	Total Carbohydrates [g/100 g]	Total Fiber [g/100 g]	Humidity [%]	Total ORAC [µmolTX/100 g]
‘Kisiel’	1332.4 ± 78.1 * /318.3 ± 18.7	2.3 ± 0.0	1.1 ± 0.0	86.0 ± 0.0	27.0 ± 4.0	10.6 ± 1.9	60.23 ± 12.73
‘Budyn’	1093.6 ± 0.3 /261.2 ± 0.1	12.0 ± 0.3	1.6 ± 0.1	75.15 ± 0	21.3 ± 2.0	11.2 ± 2.1	57.9 ± 12.59
Groats	1167.0 ± 329.8 /278.8 ± 78.8	10.0 ± 0.1	0.6 ± 0.1	79.0 ± 12.2	7.0 ± 1.7	10.4 ± 2.0	830.65 ± 138.20
Pasta	1093.7 ± 98.5 /261.3 ± 23.5	14.2 ± 2.6	2.8 ± 0.4	71.12 ± 4.7	2.5 ± 0.0	11.9 ± 1.8	184.39 ± 18.43
Gluten-free bread	891.0 ± 3.9 /213.8 ± 0.9	4.3 ± 0.3	1.1 ± 0.0	49.6 ± 0.0	2.7 ± 0.2	45.1 ± 2.3	210.34 ± 0.0
Rice wafers	1519.9 ± 0.6 /363.1 ± 0.1	9.4 ± 0.3	2.7 ± 0.1	81.9 ± 0.0	4.7 ± 0.4	6.0 ± 1.3	90.33 ± 23.35
Corn snacks	1339.9 ± 74.6 /320.1 ± 17.8	9.2 ± 0.45	0.3 ± 0.0	82.2 ± 3.39	5,9 ± 0.0	8.3 ± 1.7	312.7 ± 12.18

* Results are presented as average value ± standard deviation.

**Table 2:** The forms of iron, and ferritin loss in the designed product.

		The Content of Iron [mg/100 g]			Ferritin Loss [%]
Studied Sample		Fe (II)	Fe(III)	Fe Non-Ionic	
FSS		140.12 ± 42.31	<0.1	420.54 ± 58.01	-
Kisiel	NF	0.23 ± 0.19	0.38 ± 0.27	0.41 ± 0.3	-
	F	1.36 ± 0.43	0.38 ± 0.23	17.95 ± 0.77	0 ^a^**
Budyn	NF	0.22 ± 0.07	0.38 ± 0.13	3.06 ± 0.41	-
	F	4.23 ± 1.18	0.57 ± 0.15	11.23 ± 0.31	10 ^c^
Groats	NF	<0.1 *	<0.1	0.80 ± 0.67	-
	F	1.70 ± 0.10	0.23 ± 0.19	8.00 ± 1.00	7 ^c^
Raw pasta	NF	1.30 ± 0.60	<0.1	4.20 ± 0.70	-
Raw pasta	F	3.10 ± 0.10	<0.1	12.10 ± 0.60	3 ^b^
5′ cooked pasta	F	5.30 ± 0.10	<0.1	9.70 ± 1.40	32 ^d,e^
7′ cooked pasta	F	5.70 ± 0.70	<0.1	9.20 ± 0.18	39 ^e^
10′ cooked pasta	F	5.00 ± 0.77	<0.1	9.30 ± 1.10	37 ^e^
Water from pasta cooking 0′		0.04 ± 0.07	<0.1	0.02 ± 0.08	-
Water from pasta cooking 5′		0.02 ± 0.06	<0.1	0.02 ± 0.16	-
Water from pasta cooking 7′		0.03 ± 0.03	<0.1	0.02 ± 0.06	-
Water from pasta cooking 10′		0.02 ± 0.01	<0.1	0.02 ± 0.02	-
Bread	NF	0.7 ± 0.07	<0.1	0.80 ± 0.02	-
	F (sourdough)	5.90 ± 0.06	0.05 ± 0.04	10.90 ± 0.26 ^a^	25 ^d^
	F (yeast)	6.14 ± 0.43	<0.1	10.35 ± 0.07 ^a^	30 ^d^
Crispbread	NF	0.41 ± 0.01	<0.1	0.90 ± 0.06	-
	F	7.90 ± 0.24	3.35 ± 0.16	0.30 ± 0.03	100 ^f^
Corn snacks	NF	4.56 ± 0.02	<0.1	1.07 ± 0.25	-
	F (coarse fraction)	11.55 ± 0.13	0.08 ± 0.04	13.03 ± 0.45 ^b^	27 ^d^
	F (fine fraction)	12.77 ± 0.24	<0.1	11.66 ± 0.43 ^a^	35 ^e^
Rice wafers	NF	1.80 ± 0.18	<0.1	2.30 ± 0.25	-
	F (coarse fraction)	3.13 ± 0.25	0.2 ± 0.02	10.05 ± 0.66 ^a^	3 ^b^
	F (fine fraction)	2.75 ± 0.22	1.00 ± 0.13	9.96 ± 0.67 ^a^	4 ^b^

NF—not fortified product, F—fortified product; * <0.1—LOD for iron; **—product for which the content of ferritin iron is marked with different letters ^a–f^ differ statistically significantly at the level *p* < 0.05; results are presented as average value ± standard deviation.

## Data Availability

Data will be available on inquiry sent to the corresponding author.

## Abbreviations

The following abbreviations are used in this manuscript:

ID	Iron deficiency
IBD	Inflammatory bowel disease
FSS	Fortified soy sprouts
F	Fortified
NF	Not fortified
